# Management of massive cerebral infarction as a complication of post-tonsillectomy and analysis of the risk factors: a case report

**DOI:** 10.1186/s12883-022-03040-2

**Published:** 2023-01-05

**Authors:** Jinghao Zhao, Yubo Lei, Na Hu, Lang Xia, Liyang Zeng, Yongheng Zhang, Wei Qin

**Affiliations:** 1 Department of Intervention center, Suining central hospital, No. 127 Desheng West Road, Chuanshan District, Sichuan 629000 Suining, China; 2 Department of Radiology, Suining Central Hospital, 629000 Suining, Sichuan China; 3 Department of Cardiovascular surgery, Suining central hospital, Suining central hospital, No. 127 Desheng West Road, Chuanshan District, Sichuan 629000 Suining, China

**Keywords:** Tonsillectomy, Bleeding, Cerebral infarction, Case report

## Abstract

**Background:**

The majority of postoperative complications of tonsillectomy are bleeding. However, massive cerebral infarction following haemostasis is a very rare and serious complication and has rarely been reported clinically.

**Case presentation:**

We performed a left tonsillectomy on a patient with chronic tonsillitis. After that, active bleeding was found under the tonsillar fossa, so an exploratory hypopharyngeal haemostasis was performed. However, the bleeding worsened intraoperatively, so the patient was converted to a cervical angiographic embolization. The interventional procedure was completed successfully without an ectopic embolic event. After the procedure, the patient was transferred to the intensive care unit (ICU) and was diagnosed with acute massive cerebral infarction in the left cerebral hemisphere after awakening symptoms combined with cranial computed tomography angiography (CTA) results. Symptomatic treatment such as sedation and analgesia, dehydration to lower intracranial pressure, and maintenance of respiratory and circulatory stability was then administered. After treatment, the patient’s condition stabilized and he was transferred to the rehabilitation physiotherapy unit for rehabilitation.

**Conclusion:**

Post-tonsillectomy haemorrhage can be augmented with a carotid arteriogram to clarify whether the tonsillar fossa is at a safe distance from the posterior internal carotid artery. Furthermore, interventional haemostasis can also be performed as early as possible to reduce the incidence of complications in cases of persistent post-tonsillectomy bleeding.

## Background

Tonsillectomy is one of the most frequently performed otorhinolaryngology surgical procedures. Haemorrhage is a common and most serious complication of tonsillectomy, with an incidence of approximately 5% [1]. Haemostasis after tonsillectomy remains a challenge due to the ample arterial supply to the palatine tonsil and its origin in the main branches of the external carotid artery such as the facial and lingual arteries [2]. It is also the most common cause of prolonged postoperative hospital stay, which can be life-threatening in severe cases [3].

We reported a case of a 57-year-old female patient who underwent a left tonsillectomy for chronic tonsillitis and had recurrent postoperative bleeding from the tonsillar fossa and developed a large left-sided cerebral infarction. We further analysed the possible causes of this condition.

## Case presentation

**Fig. 1:**
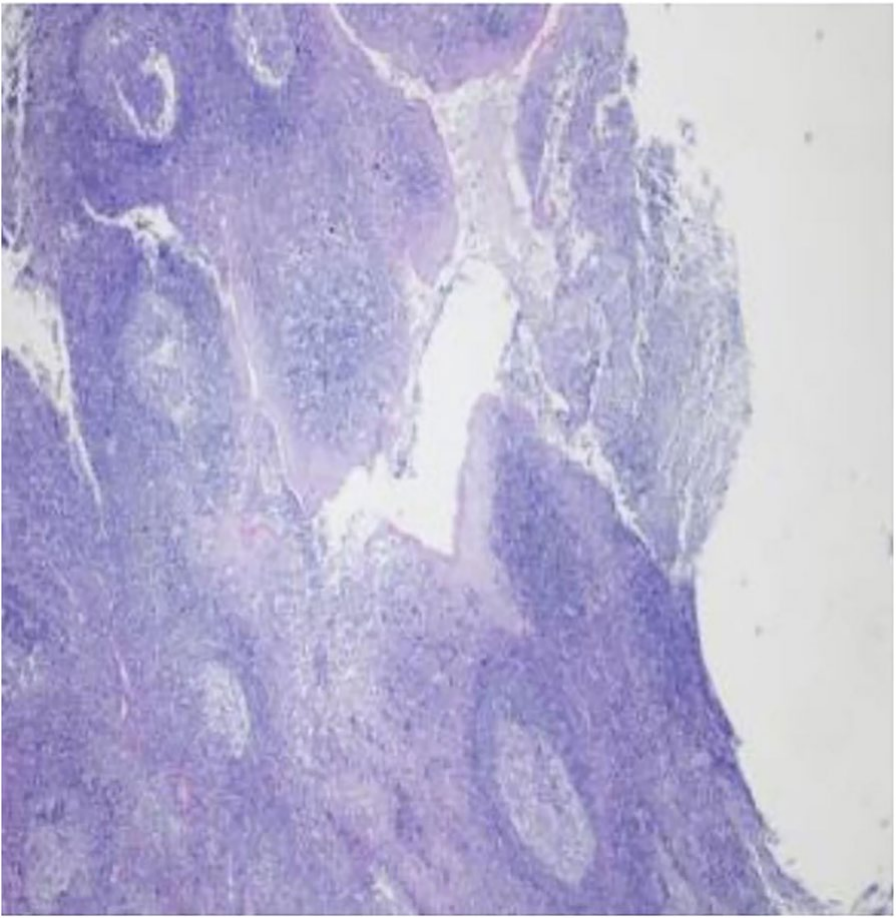
Pathological figure in the reported patient with chronic tonsillitis

The reporting of this study conforms to the CARE guidelines [4]. A 57-year-old female was admitted to our hospital for treatment of recurrent sore throat for more than 10 years. On the admission physical examination, the patient’s pharyngeal mucosa was red and moist and the soft palate mucosa was not hypertrophic or flaccid. Moreover, the uvula was moderate and lymphatic follicles were visible in the pharyngeal wall. The right tonsil was in the I level (The tonsil was located in the tonsillar fossa, which did not extend beyond the edge of the tonsillar fossa, that is, the palatoglossal and palatopharyngeal arches at the edge of the tonsillar fossa) and the left tonsil is in the II level (The size of the tonsils exceeded the size of the tonsillar fossa, but did not reach the midline of the pharynx, the level of the uvula.). There was no discharge on the surface of the tonsils and the tonsillar fossa was clean. We thus diagnosed the patient with chronic tonsillitis. The patient was in good health, no history of hypertension, no history of diabetes, no history of heart disease, no history of cerebrovascular disease, not taking anticoagulants, no abnormalities in blood glucose and lipid tests after admission. The patient had no history of bleeding and her preoperative coagulation tests were: PT: 12.2s (normal value: 11-14.5); APTT: 55.6s (normal value: 26–45); INR: 0.89 (normal value: 0.8–1.5); fibrinogen: 2.45 (normal value: 2–4); D-dimer: 0.03 (normal value: 0.01–0.5). The surgeon then performed a left tonsil plasmapheresis with the patient under general anaesthesia. The operation was completed successfully. The patient’s pathology showed chronic inflammation of the tonsils (Fig. 1).

However, the patient suffered from sore throat, painful swallowing and bloody discharge in the mouth after the procedure was performed. And the surgeon examined her and found active haemorrhages extremely visible under her left tonsillar fossa. A second hypopharyngeal exploration was performed under general anaesthesia to stop the haemorrhage. During this second pharyngeal exploration, the bleeding worsened, and the patient was then referred for interventional haemostasis, i.e. cervical angiographic embolisation, during the procedure.

**Fig. 2:**
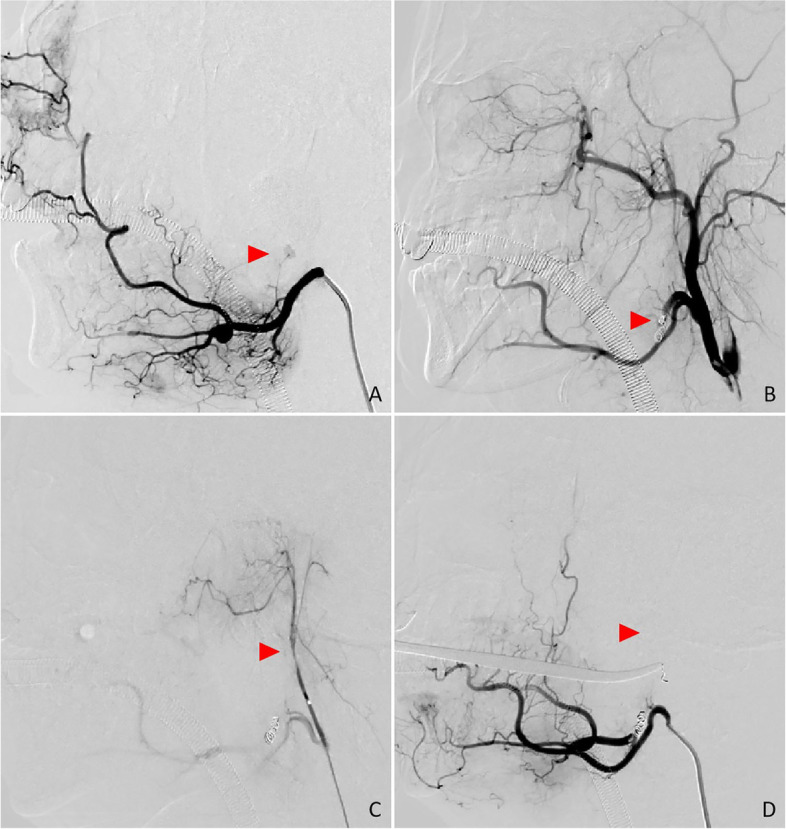
** A**. Contrast spillage was evident at the inferior border of the tonsillar fossa, as seen by the arrow, consistent with the procedure. **B**. No further bleeding was seen after the spring-ring embolization is administered. **C** and **D** Embolization of the ascending pharyngeal artery using the spring-ring

**Fig. 3:**
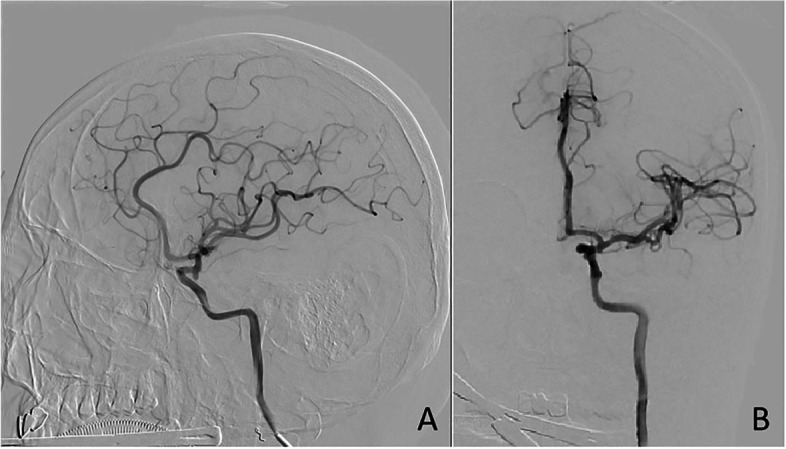
** A** and **B** showed no occlusion of cerebral vessels on the left lateral cerebral blood angiogram

A cervical angiogram showed bleeding in the patient’s left facial artery, which was embolized with a microspring coil (2–3 mm Boston Scientific Corporation, US). The patient’s ascending pharyngeal artery showed no significant bleeding and was embolized with a mini-spring coil (2 mm Boston Scientific Corporation, US) (Fig. 2). After embolization, the patient’s left internal and external carotid arteries were imaged. The angiogram showed no abnormalities in the left anterior cerebral artery, middle cerebral artery, or posterior cerebral artery (Fig. 3).

**Fig. 4:**
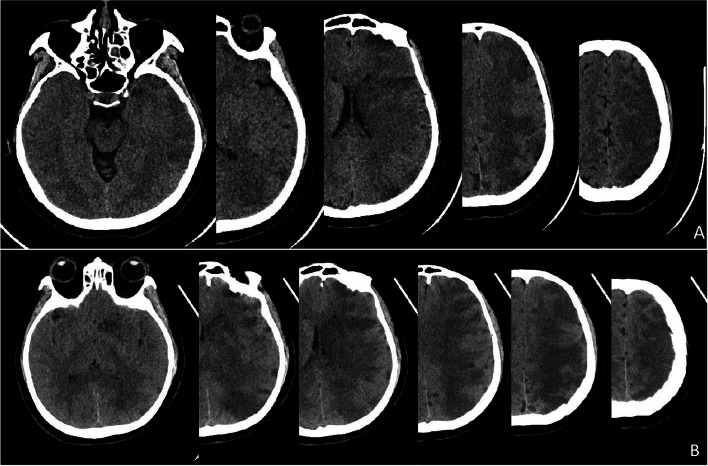
** A**. A repeat CT 12 h after surgery suggested a large cerebral infarct on the left side of the brain. **B**. A repeat CT on the 1st day after surgery suggested increased infarct edema

**Fig. 5:**
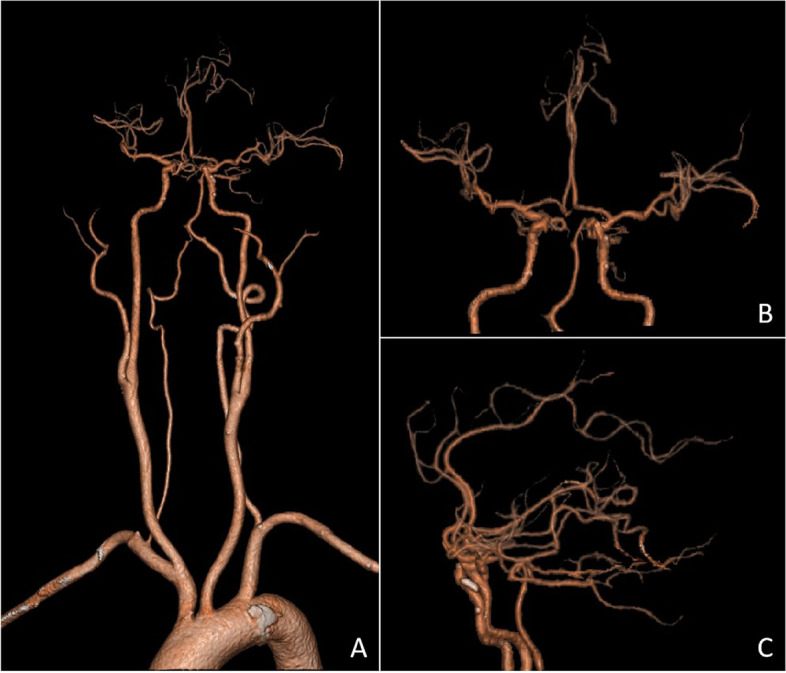
CTA of the head and neck was repeated 12 h after surgery. **A**. suggested a plaque in the left internal carotid artery, but no significant stenosis. **B** and **C** the middle, anterior and posterior cerebral arteries were not occluded as shown in frontal and lateral CTA of the head

The interventional procedure was completed successfully and the patient did not experience any ectopic embolic events. She was transferred to the ICU unit. Four hours after the procedure, the patient awoke from general anaesthesia but presented with symptoms such as bilateral eye gaze to the left, immobility of the right limb and unresponsiveness to painful stimuli. In response, the doctors considered that the patient might be suffering from an acute cerebral infarction. An urgent cranial computed tomography angiography (CTA) revealed: (1) a large, slightly hypointense shadow in the left cerebral hemisphere with slight swelling of the surrounding brain parenchyma; (2) a mixed plaque formation in the C4-6 segment of the left internal carotid artery, with no significant stenosis of the vessel. Therefore, the physician diagnosed the patient with acute massive cerebral infarction in the left cerebral hemisphere (Figs. 4 and 5). After massive cerebral infarction, patients were given ventilator assisted respiration, analgesia, sedation, brain protection and calming treatment, stress relief, moderate dehydration, intracranial pressure reduction, expectoration, organ function maintenance, blood glucose and blood pressure monitoring, circulation and internal environment stability maintenance, coagulation dysfunction correction, nutritional support, gastrointestinal motility promotion, intestinal flora improvement, prevention of deep vein thrombosis and other treatments.

**Fig. 6:**
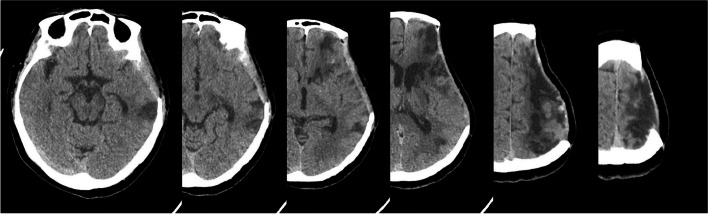
Cranial CT performed 53 days after the operation

This patient’s condition was variable and treatment was complex. This report summarised the main treatment modalities and time points of change in his condition following surgery (Table 1). And a repeat CT was performed on the 53rd day after the operation (Fig. 6).

**Table 1:** Time points of change in condition and main treatments

Timepoints	Status	Treatment
Post-operative day 1	Massive cerebral infarction	Symptomatic treatment such as sedation and analgesia, dehydration to lower intracranial pressure and maintain respiratory and circulatory stability
Post-operative day 3	Cerebral hernia	Decompression with debridement flap
Post-operative day 9	Left epidural haematoma	Left epidural haematoma removal
Post-operative day 17	Left frontotemporoparietal epidural haematoma	Left fronto-temporoparietal epidural haematoma removal
Post-operative day 41	Discontinuation of ventilator and removal of tracheal tube	Oxygen via nasal cannula
Post-operative day 42	Lung infections	Antibiotic symptomatic treatment
Post-operative day 47	Septicemia	Antibiotic symptomatic treatment
Post-operative day 51	Urinary tract infections	Antibiotic symptomatic treatment

Two months after the operation, the patient’s status was stable and she was transferred to the rehabilitation physiotherapy unit for rehabilitation. Physical examination showed that the patient was conscious and slept well, but was incontinent. Special physical examination: wheelchair is pushed into the ward with clear mind, can be pronounced, and has no words. Advanced nerve function examination was uncooperative. The pupils were equal in size and circle, with a diameter of about 3 mm. They were sensitive to light reflex. The binoculars moved normally in all directions. No nystagmus was induced. The tongue did not extend. The right nasolabial groove became slightly shallow, and the neck was soft without resistance; The muscle strength of the right upper limb is grade 0, and the muscle strength of the right lower limb is grade 3. The physical examination of deep and shallow senses and ataxia do not cooperate. The muscle tension of the right limb is reduced, the right tendon reflex (+), ankle clonus (-), negative pathological signs on both sides, and negative meningeal stimulation signs. The patient’s trunk muscle strength has improved and was able to maintain sitting or standing balance at rest without external forces. Furthermore, she was able to maintain balance while performing activities with no movement on the support surface (sitting or standing position). Her articulation has increased, her unresponsiveness was slightly better and she can wave when she saw friends.

## Discussion

Bleeding is an important complication of tonsillectomy, and the study on the complications of tonsillectomy has been a hot spot in clinical work [5]. However, at present, massive cerebral infarction following haemostasis is a very rare and serious complication and has rarely been reported clinically.

**Fig. 7:**
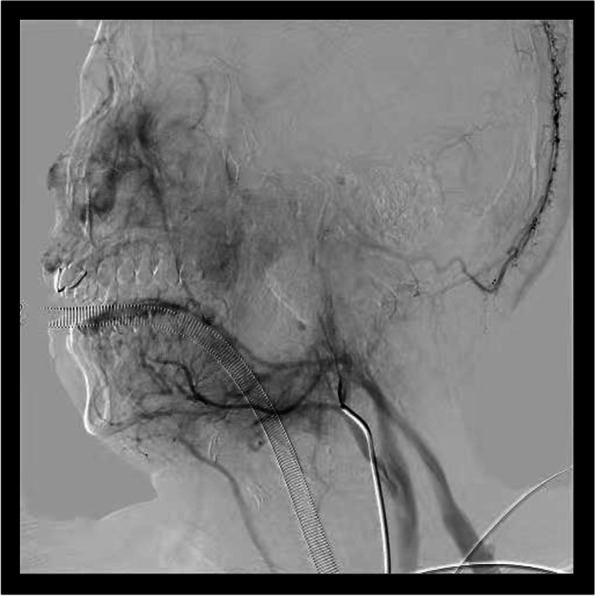
During the intraoperative cerebral angiography, we can observe that the drainage vein was developed smoothly without thrombosis

**Fig. 8:**
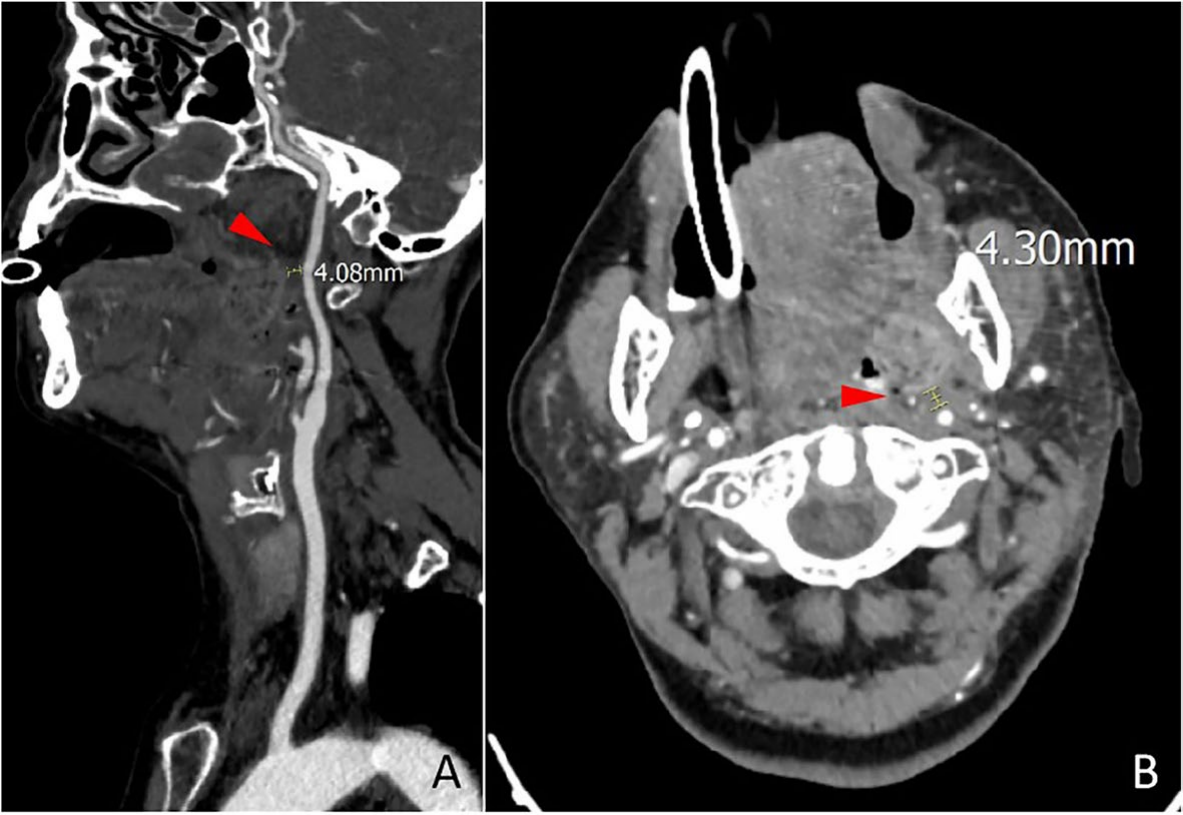
As indicated by the arrows in figures **A** and **B**, the tonsillar fossa in the operative area was only 4 mm from the left internal carotid artery and formed a pressure point with the posterior atlas, causing compression of the internal carotid artery

**Fig. 9:**
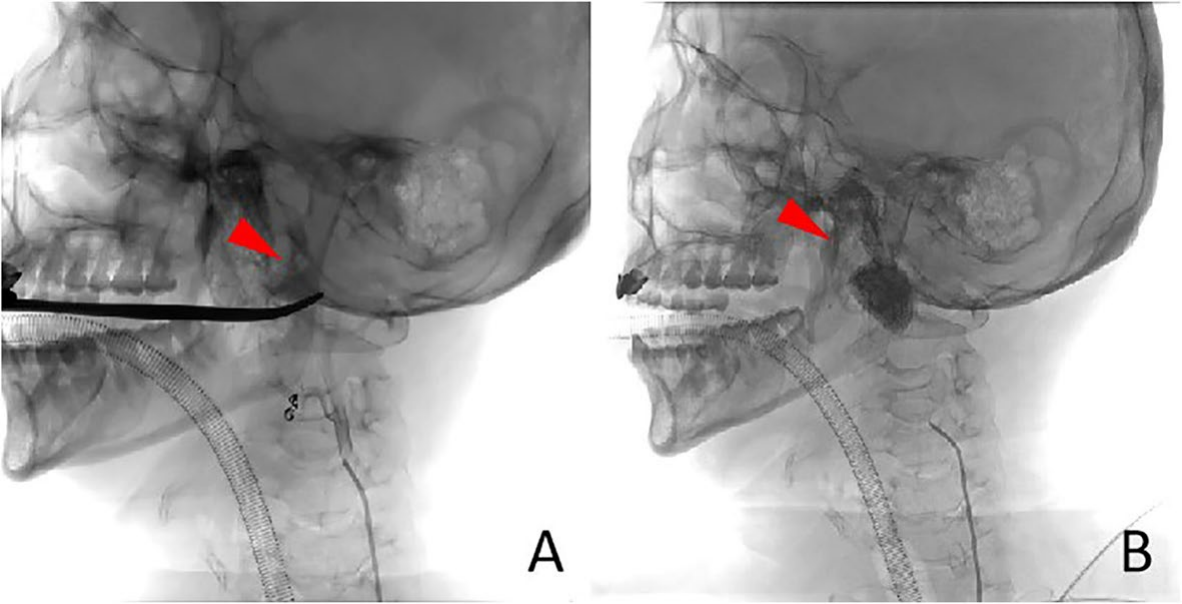
The arrows in figures **A** and **B** indicated that intraoperative hemostatic forceps and tonsillar fossa filling objects can compress the internal carotid artery

In this case, the patient had a medical history of 10 years, and the left tonsil was II ° enlarged, besides, the patient often feels uncomfortable in the throat, swallows with foreign body feeling, snores at night with respiratory disorder. Hence, we chooses surgery, rather than pursuing medical management. The reported patient presented with acute massive cerebral infarction after a second general anaesthetic pharyngeal exploration for haemostasis combined with interventional haemostasis. The attending physician is an attending physician with 22 years of experience in tonsillectomy. During hemostasis, he did not press for a long time. Therefore, we first ruled out cerebral infarction caused by prolonged hemostasis and compression during operation. Secondly, we ruled out the possibility of jugular vein thrombosis. During the intraoperative cerebral angiography, it was observed that the drainage vein was well developed without thrombosis **(**Fig. 7**)**. After the operation, the hospital organized several joint multidisciplinary consultations (MDT) to analyse the possible causes of the cerebral infarction, which mainly included the following: (1) interventional ectopic embolization. A spring coil was used as the main embolic material during the interventional procedure, and the coil embolized the main stem of the vessel. After the embolization procedure, the patient also underwent left-sided cerebral angiography which did not reveal any abnormalities, and the imaging manifestations of interventional ectopic embolism were mostly small randomly distributed cerebral infarcts characteristic of the patient, which were also not consistent with a large cerebral infarction, so it was excluded. (2) The intraoperative cervical plaque was dislodged, and the postoperative CTA suggested a mixed plaque in the C4-6 segment of the left internal carotid artery, and the CT showed mostly small plaques. Small plaque detachment was not enough to cause a large cerebral infarction, so it was excluded. (3) Anesthesia accident. The patient’s entire operation lasted for more than 10 h, and he lost about 900 ml of blood during the whole monitoring process, with about 2000 ml of fluid input. The heart rate was maintained at a stable level, the mean arterial pressure was maintained at about 70 mmHg, and the vital signs were basically stable, so it was excluded. (4) As a result of the tonsil surgery, the patient’s condition was characterized by a large cerebral infarction, but neither the postoperative digital subtraction angiography (DSA) angiogram nor the postoperative CTA of the left cerebral vasculature showed any significant abnormality in the vessels. The cerebral infarction event should have occurred during the tonsil surgery and should have been a post-compression revascularization to explain why the cerebral infarction event occurred without any abnormality in the angiogram. The patient had undergone a plasma tonsillectomy and the tissue gap between the tonsillar fossa and the internal carotid artery was already very close to the nearest 4 mm due to repeated bleeding and two postoperative debridements, and the artery was posterior to the cervical spine, which was vulnerable to the formation of a stress point (Fig. 8). Intraoperative hemostatic forceps or tamponade compression to stop bleeding would most likely result in temporary compression of the internal carotid artery, and occlusion of the internal carotid artery for more than 10 min could cause irreversible loss of brain tissue (Fig. 9).

## Conclusion

Bleeding is a common complication after tonsillectomy, and the development of a large cerebral infarction with repeated probing to stop bleeding was unpredictable, but fortunately, the patient has passed from the danger phase and was actively rehabilitating. In this study, we got some experience about the diagnosis and treatment of this patient. First, post-tonsillectomy haemorrhage can be augmented with a carotid arteriogram to clarify whether the tonsillar fossa is at a safe distance from the posterior internal carotid artery. Secondly, intractable post-tonsillectomy haemorrhage can be stopped by early intervention after surgery to reduce the complication rate. Thirdly, for patients with intractable bleeding after tonsillar surgery, we should actively carry out multidisciplinary joint consultation, especially in the hematology department, intervention center and other related departments. If this patient chooses interventional hemostasis instead of surgical hemostasis after the first debridement and hemostasis, the outcome may be different.

## Data Availability

All the data generated and/or analyzed during this study are included in this published. article.

## Abbreviations

CTA: computed tomography angiography
MDT: multidisciplinary consultations
DSA: digital subtraction angiography
